# Book Notices

**Published:** 1883-09-20

**Authors:** 


### *BOOK NOTICES.*

Transactions of the Medical Society of Pennsylvania, held at Norristown, May 1883; 509 octavo pages, bound in cloth.

Officers elect for 1884: President—Henry H. Smith.

Vice-Presidents: 1st. Ellis Phillips, Schuylkill Co. 2nd. H. B. Van Balzah, Clearfield Co. 3d. J. W. Kerr, York Co. 4th S. S. Schultz, Montour Co.

Secretaries: Wm. B. Atkinson, Philadelphia; Morris S. Fouch, Philadelphia; John S. Lee, Philadelphia.

Treasurer: Benjamin Lee, Philadelphia. Wm. B. Atkinson, Chairman of Committee of Publication.

Many able and instructive papers are contained in this book on various subjects of interest which we have not space to detail. We have always found the Pennsylvania transactions large, neatly bound and valuable. The next meeting is appointed for Philadelphia, the first Wednesday in May, 1884.

Transactions of the Michigan State Medical Society for the year 1883, held at the Academy of Music, Kalmazoo, Michigan, May 9th and 10th.

The address of welcome was by E. W. DeYoe, and the President's address by G. W. Topping, M. D. Papers were presented by—

Dr. Wm. Brodie, on *Tumors of the Scalp.* Dr. C. J. Lundy, on *Errors of Refraction.* Dr. A. R. Smart, on *Foreign Body in the Ear.* Dr. Eugene Smith, on *Ulcers of the Cornea.* Dr. H. J. Reynolds, on *Urethral Inflammation.* Dr. H. B Baker, on *Epidemic Waves of Diphtheria* Dr. T. N. Reynolds, on *Timely Catharsis.* Dr. J. A. Post, *Water in Health and Dioease* Dr. E B. Ward, on *Pro bono Professions.*

Officers elect: A. F. Whelan, of Hillsdale, President. Horace Tupper, of Bay City, 1st Vice-President J. S. Hamilton, of Tecumseh, 2d Vice-President. H. B. Barnes, of Ionia, 3d Vice-President. Augustus Kaiser, of Detroit, 4th Vice-President. Geo. E Ranney, of Lansing, Secretary A. R. Smart, of Hudson, Treasurer.

The transactions are neatly gotten up The papers and addresses are creditable, and bespeak a good degree of zeal and interest in the Society and in the profession. The next meeting will be at Grand Rapids, in May, 1884 We make this suggestion to Secretaries of societies: Always designate the time and place of next meeting in a note appended to the list of officers elect, so that it may be easily found.

